# Directions of Deep Brain Stimulation for Epilepsy and Parkinson’s Disease

**DOI:** 10.3389/fnins.2021.680938

**Published:** 2021-06-14

**Authors:** Ying-Chang Wu, Ying-Siou Liao, Wen-Hsiu Yeh, Sheng-Fu Liang, Fu-Zen Shaw

**Affiliations:** ^1^Department of Computer Science and Information Engineering, National Cheng Kung University, Tainan, Taiwan; ^2^Institute of Basic Medical Science, National Cheng Kung University, Tainan, Taiwan; ^3^Institute of Medical Informatics, National Cheng Kung University, Tainan, Taiwan; ^4^Department of Psychology, National Cheng Kung University, Tainan, Taiwan

**Keywords:** deep brain stimulation, PD, epilepsy, closed-loop, open loop

## Abstract

**Background:**

Deep brain stimulation (DBS) is an effective treatment for movement disorders and neurological/psychiatric disorders. DBS has been approved for the control of Parkinson disease (PD) and epilepsy.

**Objectives:**

A systematic review and possible future direction of DBS system studies is performed in the open loop and closed-loop configuration on PD and epilepsy.

**Methods:**

We searched Google Scholar database for DBS system and development. DBS search results were categorized into clinical device and research system from the open-loop and closed-loop perspectives.

**Results:**

We performed literature review for DBS on PD and epilepsy in terms of system development by the open loop and closed-loop configuration. This study described development and trends for DBS in terms of electrode, recording, stimulation, and signal processing. The closed-loop DBS system raised a more attention in recent researches.

**Conclusion:**

We overviewed development and progress of DBS. Our results suggest that the closed-loop DBS is important for PD and epilepsy.

## Introduction

Deep brain stimulation (DBS) has been used since the 1980s for treatment of movement disorders. DBS has several apparent advantages over lesion therapy. It is reversible and provides superior symptom relief with fewer complications than lesions. DBS creates maximal efficacy by adjustment of treatment parameters after implantation and can be applied bilaterally while bilateral lesions usually lead to a high risk of side effects (Okun and Vitek, 2004). First used for Parkinson’s disease, DBS is an FDA-approved treatment for Parkinson’s disease (PD), essential tremor, and dystonia. It is estimated that DBS devices have been implanted in ∼150,000 patients with movement disorders of the United States (Benabid et al., 1987). This success has encouraged the use of DBS across a broad range of neuropsychiatric disorders. More recently, DBS has been approved for obsessive-compulsive disorder and medically refractory epilepsy. Effect of clinical trials studying the use of DBS for the treatment of major depression (Dandekar et al., 2018) and Alzheimer’s disease (Lozano et al., 2016) have limited because of inconsistent outcomes for the majority of the aforementioned neuropsychiatric disorders. Several critical aspects of therapy remain unsolved, in particular, how, where and when stimulation should be delivered according to individual anatomical and pathophysiological differences. This review addresses these factors on patients with epilepsy or Parkinson’s disease.

PD typically develops between the ages of 55–65 years. Approximately 0.3% of the general population is affected. Parkinson’s disease is a neurodegenerative syndrome involving multiple motor and non-motor neural circuits in the basal ganglia (Lang and Lozano, 1998). Motor manifestations of the disorder commonly include a resting tremor, rigidity (stiffness), slowness of movements (bradykinesia), and shuffling steps. In addition to these classical symptoms, PD also has a multitude of non-motor manifestations, including disturbance of mood (e.g., depression, anxiety), cognition (dementia and frontal-lobe dysfunction), and autonomic dysfunction (e.g., sexual dysfunction or digestive problems). DBS is one of the most effective treatments for advanced PD. Conventional DBS using an open loop architecture targets at the subthalamic nucleus (STN) or globus pallidus interna (GPi) which provides, on average, only 40% improvement in the motor items. Paradoxically, DBS of the STN can worsen motor function by not only influencing pathological but also physiological neural activity (Chen et al., 2006). The potential of conventional DBS is often limited due to stimulation induced side effects. More alternative technologies have been suggested to minimize the worse complications of DBS in PDs.

Epilepsy is a common chronic neurological disorder characterized by spontaneous recurrent seizures and affects around 60 million patients worldwide (Engel, 2016). As many of 40% of these patients have drug-resistant epilepsy (DRE). The international League Against Epilepsy has proposed that DRE can be defined as a failure of adequate trials of at least 2 antiepileptic drugs that are appropriately chosen, used, and tolerated. Approximately 1 million people in the US continue to have seizures despite adequate treatment with antiseizure drugs, and DRE can be associated with severe disability and morbidity. The incidence of sudden unexpected death in epilepsy is higher in patients with medically resistant epilepsy than in a general population (Harden et al., 2017). The first-line treatment for DRE is respective surgery. However, when surgery is contraindicated or ineffective, DBS has emerged as an important treatment option. DBS involves the delivery of a predetermined (open-loop) program of electrical stimulation to deep brain structures via implanted electrodes connected with a pulse generator. DBS of the anterior nucleus of the thalamus (ANT) has been approved for the treatment of refractory epilepsy. Surgical (e.g., infection, hemorrhage and pain) and stimulation-related (e.g., headache, sleep disturbance and increased anxiety or depression) adverse effects are similar to those observed from DBS for movement disorders (Fisher and Velasco, 2014). Compared to PD, spontaneous seizures occur in some unexpected scenarios and are not continuous events to perform an open-loop DBS stimulation in patients with epilepsy due to several side effects. Prevalence of side effects strongly depends on the target nucleus and the anatomy and functionality of the surrounding tissues. As such, more commercial DBS device for PDs use an open-loop architecture (Table 1), and closed-loop configuration is highly selected for epilepsy (Table 2).

**TABLE 1:** Open-loop neural stimulation system.

	Medtronic	Abbott	Boston scientific	St. Jude
Device	Activa RC	Infinity 7	Vercise PC	Brio
FDA class	II	III	II	II
Volume (cm^3^)	22.0	38.6	33.0	22
Stimulation site	DBS	DBS	DBS	DBS
Application	PD, Epilepsy	PD	PD	PD
Stimulation channels	8	16	16	16
Frequency (Hz)	2–250	2–240	2–250	2–240
Pulse width (μs)	6–450	20–500	20–450	50–500
Battery longevity (years)	9	4–5	15(US)–25(EU)	10
Battery type	Rechargeable	Non-rechargeable	Rechargeable	Rechargeable
MRI-compatible	Yes	Yes	Yes	No
Intensity	0–25.5 mA/0–10.5 V	0–12.75 mA	0–20 mA	0–12.75 mA
Data monitoring	Wireless	Wireless	Wireless	Wireless

*DBS, Deep brain stimulation; N/R, Not Reported; PD, Parkinson’s disease.*

**TABLE 2:** Closed-loop neural stimulation system.

	Neuropace	Medtronic	LivaNova
Device	RNS system	Activa PC + S	Aspire SR
FDA class	III	II	II
Volume (cm^3^)	12.94	37.0	N/R
Stimulation site	DBS	DBS	Vagus Never stimulation
Application	Epilepsy	PD	Epilepsy
Stimulation channels	8	8	N/R
Frequency (Hz)	1–333	2–250	1–30
Pulse width (μs)	40–1,000	60–450	130–1,000
Battery longevity (years)	2–3.5	3–5	4–7
Battery type	Non-rechargeable	Non-rechargeable	Non-rechargeable
MRI-compatible	No	Yes	Yes
Intensity	0.5–12 mA	0–25.5 mA/0–10.5 V	Current/0–3.5 mA
Data monitoring	Wireless	Wireless	Wireless

*DBS, Deep brain stimulation; N/R, Not Reported; PD, Parkinson’s disease.*

**FIGURE 1:**
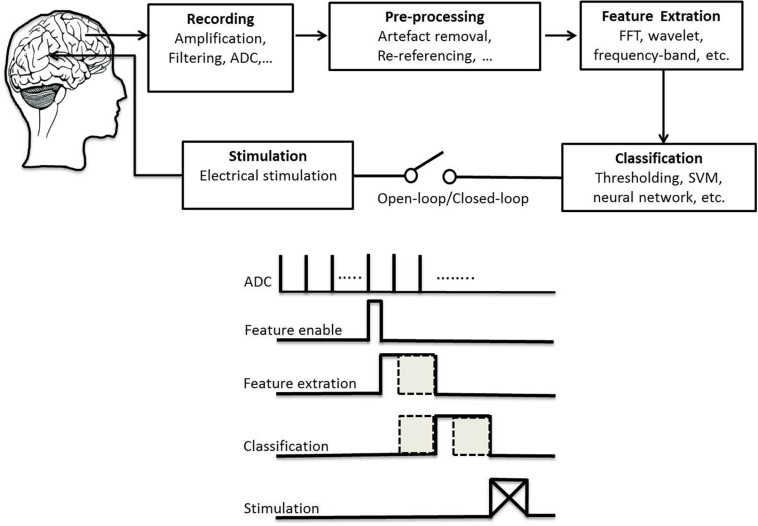
Schematic plot of a technical system for deep brain stimulation **(top)** and its flowchart of signal processing **(bottom)**.

High-frequency DBS was thought to function as a reversible lesion by inhibiting neurons near the stimulating electrode (Laxpati et al., 2014). However, it has advantages over ablation including its reversibility, the ability to adjust stimulation setting to optimize efficacy and minimize side effects, the ability to perform bilateral procedures safely, and low risk of cognitive problems (Yu and Neimat, 2008). A critical aspect of DBS efficacy is patient selection and the appropriate target location based on patient’s symptom profile, age and cognitive status. These choices greatly depend on the expertise of the surgical team and vary from center to center. Up to 50% of implanted patients can experience stimulation-induced side effects (Volkmann et al., 2009). Emerging technologies aim to minimize these side effects and increase efficacy of DBS. We review studies that provide alternative strategies to state-of-the-art DBS with different control policies. We emphasize important considerations for therapy safety that continuously adapt stimulation parameters according to a disease biomarker in a closed-loop configuration with a higher detection rate. In general, a DBS technology system consists of several components (Figure 1): electrical stimulation and required aspects of the closed-loop architecture (including recording, preprocessing, feature extraction, classification). This study reviews existing research efforts in signal acquisition, biomarker algorithms, and system integration to provide a solid foundation toward the future development of smart and fully embedded integrated circuits.

## DBS System Oerview

A DBS system is composed of one or more electrode leads implanted in the brain and extension wires tunneled underneath the skin to an implanted pulse generator (IPG) positioned below the collar bone (Miocinovic et al., 2013). There are two main functions of IPG devices: neural recording and stimulation. Owing to the extremely low amplitude of EEG signals, low-noise and low-power electronic design are necessary for recording and analysis (Ranjandish and Schmid, 2020). Subsequently, we introduce DBS electrodes technology, neural recording amplifier and electrical stimulation system in the following paragraphs.

### Electrodes for DBS

Development of DBS electrodes for implantation into the human brain began in the mid-twentieth century in the interest of treating movement-related disorders. The crucial characteristics of an electrode include biocompatibility, inertness, durability, stability over time, surgical feasibility, good conductivity, electrical properties, tractability, appropriate current delivery and spatial configuration. The standard DBS electrode configuration consists of 1.27 mm diameter cylinder with four stimulating electrode contact. Each cylindrical contact is 1 mm in length and 0.5–1.0 mm pitch (Medtronic, Inc.). Each active electrode can emit a continuous spherical electric field radiating outward from the stimulation site. In recent years, several new electrode designs have been proposed allowing to arrange the electrical field perpendicular to the lead as a directional DBS (Steigerwald et al., 2019). For example, more simple models split up the conventional ring contacts in 3–4 segments spanning 90° or 120° to create horizontal current flow. That could modify the current threshold for beneficial and adverse effects, which depends on whether current is injected toward or away from the underlying anatomical structure (Anderson et al., 2018). Industry and clinicians hoped that the directional DBS would reduce the risk of stimulation-induced adverse effects and optimize the clinical benefit of DBS. These segmented electrodes allow clinicians to modify side-effect thresholds and create a greater margin between symptom suppression and side-effect induction (Dembek et al., 2017).

Once a target is determined, the accuracy of stimulated site is critical so that the volume of tissue activated matches the target structure as best as possible. In addition to directional electrode design, thin-film planar arrays could provide further improvement to spatial specificity of stimulation and recording through reduced contact size and increased contact numbers (Connolly et al., 2015). Advancements in DBS electrode technology have seen large number of electrodes used to emit a directional, rather than uniformly spherical, which allows for unique and simultaneous electrical stimulation at different contacts (Krauss et al., 2020). Compared to silicon-based thin-film array, microwires have a more stable for a long-term recording and stimulation. Recently, three-dimensional microwire arrays combined with CMOS chips are developed for chronic recording and stimulation with a greater success in a long-term treatment (Obaid et al., 2020).

However, increased electrode numbers come with trade-offs. The greater flexibility afforded by segmented electrodes and thin planar arrays considerably increases the degrees of freedom allowed in programming to increase its sensitivity or specificity for DBS. This flexibility increases the burden on the clinical team because parameter selection and optimal stimulation contact most depend on a process of trial and error. Therefore, automated or support tools for assisting clinicians in determining optimal stimulation parameters are sorely needed. For example, it has recently been shown that the use of a disease biomarker, such as heightened rhythmic neural activity, can reduce the amount of time needed for programming segmented electrodes for the PD treatment (Fernández-García et al., 2017). Strategies that consider electrode location and anatomical landmarks in conjunction with individualized neuroimaging could provide additional information needed to reduce the degrees of freedom associated with programming DBS electrodes.

Numerous kinds of materials have been used for DBS electrodes. The choice electrode technology always has strong impact on an implantable neurostimulator’s efficacy, efficiency, longevity, precision, and cost. The electrical inefficiency of platinum electrodes causes unnecessary power consumption and reduced battery lifetime. Increasing efficiency can extend implantation life and reduce battery size. Thus modern DBS implants can benefit from more efficient electrode materials (Petrossians et al., 2016). The platinum-iridium alloy expresses superior electrical properties (including minimal toxicity and excellent conduction property) and reveals wonderful mechanical robustness to insertion into brain for DBS.

Materials of the DBS electrodes and geometries are altered to attain low impedance path for charge injection, high charge transfer, and compromised spatial resolution. The electrode fabrication design parameters, e.g., shape, materials, and fabrication technique should be optimized to achieve the best electrode performance. Advantages and disadvantages of microwire or silicon array have been reviewed elsewhere (Ghane-Motlagh and Sawan, 2013). A small electrode size is required for multichannel stimulation, but this increases impedance and affects the signal-to-noise ratio. The electrode array increases insertion force during implementation into the brain. The estimated insertion force and mechanical failure modes are investigated for brittle and ductile materials (Gabran et al., 2014). The mechanical performance of the electrode array is primarily affected by the materials and geometry. A figure of merit in terms of mechanical performance, fabrication cost, geometry of the electrode, and cross sectional area of the electrode, etc., has been proposed to select the best electrode design parameters from different electrode arrays (including silicon, copper, nickel, polyimide materials; Draz et al., 2018).

In addition to different materials and geometries of the electrode, understanding electrochemical behavior of electrode materials and insulation in a long-term process is crucial (Gimsa et al., 2005). Corrosion resistance of the electrode metal is of greatest important for its long-term stability and biocompatibility. The electrode surface is not corroded uniformly because varied spatial distribution of the corrosion of metal and erosion of the plastics insulation regarding to the site dependence of the current density (Gimsa et al., 2006). Erosion of the plastics insulation is known as a severe problem, but it remains largely unknown so far.

### Neural Recording Circuits

There are two main functions of implantable biomedical devices: neural recording and stimulation. Electroencephalogram (EEG), electrocorticogram (ECoG), local field potential or action potential is often used in the closed-loop design of an DBS. Because the extremely low amplitude of EEG signals, recording system with low-noise, low-offset, high CMRR and low-power characteristics is necessary for further analysis (Ranjandish and Schmid, 2020). Two principal categories of chopping schemes with instrumentation amplifier are designed. The AC-coupled chopped instrumentation amplifier can effectively reduce 1/f noise and amplifier offset with a CMRR of > 120 dB, an input referred noise density of 57 nV/Hz, and power consumption of 60 μW (Yazicioglu et al., 2007). However, the DC-coupled amplifier limits the electrode offset to only ± 50 mV. On the other hand, a virtual ground node of the amplifier is designed to reject a large DC offset but scarifying with a CMRR of ∼60 dB (Verma et al., 2010), which is a remarkable drawback for multichannel recording. Amplifier circuits for data acquisition using the system on chip have been reviewed in detail elsewhere (Yang and Sawan, 2020).

In the closed-loop system, rejection of stimulation current-induced artifact has been emphasized. Careful placement of stimulation, recording, and reference electrodes, e.g., symmetrical configuration between electrodes, has shown to reduce the stimulation artifact of a common-mode-like signal by differential amplifier with high CMRR. A newer front-end technique has focused on mitigating the effects of stimulation artifacts by preventing saturation in a high-gain preamplifier. Alternatively, disconnecting the front-end via a series switch at the input prevent artifacts from the recording circuitry (Venkatraman et al., 2009). However, this design can suffer from slow transient settling once reconnected. More advanced technologies to achieve artifact-free recording during stimulation have been reviewed previously (Zhou et al., 2018).

### Neural Stimulation Techniques

DBS is very often to activate or inhibit neurons with implantable medical devices. The use of electrical stimulation in clinical practice requires a high degree of safety, stability, and programmability, and also takes into account the issues of voltage-power consumption and heat dissipation. There are two main modes of stimulation: current-mode and voltage-mode. Most of available commercial DBS devices offer the constant voltage stimulators due to higher power efficiency. At this moment, the magnitude of current depends on impedance between tissues and electrodes. The impedance variations in the brain tissue and the electrode-tissue interface always exist in various stages for DBS implementation. Impedance fluctuations have been observed during the first 3 months after surgery (Lempka et al., 2009). A mean 73 Ω/year reduction in impedance in most DBS electrode contacts has been recorded (Satzer et al., 2014). Unbalanced charging is more likely to happen in voltage stimulation and thus relatively lack of safety. Some “excess voltage” may result in gas evolution (e.g., hydrogen evolution at the cathode), redox reactions of organic molecules, and the deposition of potentially harmful materials (e.g., metal ions, chlorine and toxic organic products in the tissue; Gimsa et al., 2005). The constant current stimulation mode is extensively used in DBS. Adaptive currents are used to supply the stimulus current to the load. Total amount of charges injected in the current stimulation mode depend on the magnitude and duration of the stimulation current. Traditionally, the current intensity is set in the range of 0.5–15 mA for 0.3-ms pulse width. Compared with the constant voltage stimulation mode, constant current stimulation mode provides higher controllability and safety; however, lack of power efficiency is worth to be improved (Ortmanns et al., 2007; Sit and Sarpeshkar, 2007; Liu et al., 2008).

Neural stimulation is used to activate or modulate neural activity. When charge is continuously deposited onto an electrode, the resulting electric field becomes strong enough to trigger a response from neighboring neurons. The charge must be removed from the electrode to prevent build up possible permanent tissue damage. Biphasic stimulation is better for this operation rather than monophasic stimulation in DBS (Fung et al., 1998). A traditional charge balanced current stimulation has been widely used. There are at least three problems faced by this topology (Wu et al., 2018): mismatch between the two current sources, excess power consumption for supplied voltage, and large IC area consumption to support one stimulator per electrode. Each stimulator utilizes a single current source for both positive and negative stimulation phases to reduce current mismatch effect and to eliminate the need for calibration (Biederman et al., 2015). An adiabatic, charge-recycling architecture without utilizing off-chip components can minimize power consumption (Arfin and Sarpeshkar, 2011). Multiple supply voltages from DC-DC converter are utilized to minimize the power consumption throughout the stimulation cycle.

Continuous open-loop stimulation of DBS uses static stimulation parameters to measure behavioral or functional outcomes. As we can see in previous studies of PD (Table 3) or epilepsy (Table 4), continuous open-loop DBS is a popular design at the beginning. Common stimulation parameters of open-loop DBS are ≥ 100 Hz at 1–10 V for ANT stimulation for refractory temporal lobe epilepsy, ≥ 130 Hz at 1–5 V for hippocampus and STN stimulation for refractory temporal lobe epilepsy, tens to high frequency stimulation at 1–10 V for stimulation of centromedian nucleus of the thalamus for generalized tonic-clonic seizures. For PDs, 130–200 Hz at 2–5 V for STN and GPi stimulation. Cycling of 1 min on and 5 min off at 5 V with 145 Hz stimulation has been suggested for epilepsy. All stimulation parameters are designed in commercial products (Table 1). There is no difference between cycling and continuous stimulation and no association between output voltage and seizure reduction (Li and Cook, 2018). DBS-induced side effects can be reduced by minimizing the duration and intensity of stimulation or changing to bipolar mode.

**TABLE 3:** Recording and stimulation system for PD.

Source	Platform	Channel (s)	Intensity	Control	Filter (Hz)	Clinical results (reduction)
McCreery et al. (2006)	N/A	16	0–0.0265 mA	O	N/A	N/A
Boulet et al. (2006)	N/A	N/A	0–0.35	O	10–200	N/A
Fang et al. (2006)	A-M System	N/A	0–0.2 mA	O	0–130	∼80% initiating time for STN DBS.
Gubellini et al. (2006)	P2MP, Marseille	N/A	0–0.08 mA	O	0–130	N/A
Baunez et al. (2007)	N/A	4	0–0.05 mA	O	0–130	77–85% correct response
Dorval et al. (2008)	N/A	N/A	2.4–4 V	O	0–136	Burst-duration in 67% bursting cells.
Harnack et al. (2008)	PIC16C54	1	0.05–0.6 mA	O	0–131	N/A
Winter et al. (2008)	N/A	N/A	0–0.3 mA	O	0–130	N/A
Lee et al. (2008)	N/A	64	0.003–0.135 mA	C	17–5.3 k	N/A
Paulat et al. (2011)	PIC16C54	N/A	0–0.1 mA	O	N/A	N/A
Nowak et al. (2011)	N/A	1	0–0.5 mA	O	0–130	N/A
Azin et al. (2011)	N/A	4	0–0.0945 mA	C	0–10 k	N/A
Loukas and Brown (2012)	NI DAQ	1	Unipolar (0–10 V), bipolar (± 5 V)	C	13–30	N/A
Forni et al. (2012)	N/A	1	0.05–0.12 mA	O	0–130	44% (2 weeks), 48% (5 weeks) for STN DBS
de Haas et al. (2012)	PICKIT 3	1	0.02–0.1 mA	O	0–131	N/A
Lee et al. (2013)	N/A	4	0.08–2.48 mA	O	N/A	N/A
Poustinchi and Musallam (2013)	N/A	N/A	N/A	O	0.1–100	N/A
Heo et al. (2015)	MSP430F2013,	2	0–3 V	O	N/A	N/A
Parastarfeizabadi et al. (2016)	MCU	1	0–0.2 mA	C	0–130	N/A
Arlotti et al. (2016)	MSP 430	1	0–3 V	C	N/A	∼30%
Herron et al. (2016)	Activa PC + S	1	0–2.5 V	C	N/A	Tremor for 84.5% samples
Little et al. (2016)	N/A	N/A	2.7 ± 0.2 V	C	N/A	N/A
Wu et al. (2017)	IEC 60601-1	1	N/A	C	12–30	N/A
Swann et al. (2018)	Activa PC + S	8	N/A	C	60–90	Energy saving 38–45%
Chen et al. (2018)	FPGA	16	0–0.25 mA	C	30–10 k	N/A
Jia et al. (2018)	N/A	16	0–10 mA	O	N/A	N/A
Zhou et al. (2019)	WAND	128	0–5 mA	C	1–200	N/A
Fleischer et al. (2020)	N/A	N/A	0–0.3 mA	O	100–500	N/A
Xu et al. (2020)	NI DAQ	1	0–0.06 4 mA	C	1–8 k	N/A

*C, closed-loop; N/A, not available; O, open-loop.*

**TABLE 4:** Recording and stimulation system for epilepsy.

Source	Platform	Channel (s)	Clock (MHz)	Intensity	Control	Filter (Hz)	Clinical results (reduction)
Boon et al. (2001)	NCP	N/A	N/A	0–3.5 mA	O	1–143	50% in 1/3, 30–50% in 1/3 and no response in 1/3 samples
VelıìŠek et al. (2002)	A-M Instruments	N/A	N/A	0–0.28 mA	O	0–130	N/A
Cohen-Gadol et al. (2003)	VNS system	N/A	N/A	0–8 mA	O	1–70	75% in 1/5 and 50% in 35% samples
Lopez-Meraz et al. (2004)	Paxions	1	N/A	0.1–0.5 mA	O	1	N/A
Kerrigan et al. (2004)	Medtronic itrel 2	N/A	N/A	1–10 V	O	0–130	50% in 4/5 samples after 3 months
Velasco et al. (2005)	Medtronic itrel 3	N/A	N/A	0–2.28 V	O	N/A	tonic seizures:43% after 24 months,
Cuellar-Herrera et al. (2006)	Paxions	N/A	N/A	0.12–0.66 mA	O	0–130	N/A
Boon et al. (2007)	Dual screen 3628	128	N/A	N/A	O	130–200	100% in 1/10, > 90% in 1/10, ≥ 50% in 5/10, 30–49% in 2/10, no response in 1/10 samples
Avestruz et al. (2008)	N/A	4	N/A	0–0.01 mA	C	0–1 k	N/A
Halpern et al. (2009)	Medtronic itrel 2	N/A	N/A	4–5 V	C	90–130	∼45%
Boon et al. (2009)	VNS system	N/A	N/A	0–1 mA	O	N/A	40–50%, 100% in 5–10% samples
Jobst et al. (2010)	Minneapolis, MN	N/A	N/A	0.1–5 V	O	130–150	N/A
Kotagal (2011)	VNS system	N/A	N/A	0.25–3.5 mA	O	1–30 Hz	50% in 35%, 75% in 50% samples
Chen et al. (2011a)	FPGA	1	13.6	N/A	C	N/A	N/A
Chen et al. (2011b)	FPGA	4	402	N/A	C	0–3.2 k	N/A
Azin et al. (2011)	N/A	4	1	0–0.029 mA	O	0–10 k	N/A
Young et al. (2011)	CC2430	1	32	0.02–0.05 mA	C	0.8–72	N/A
Zanos et al. (2011)	Neurochip-2	3	N/A	0–5 mA	C	0.5–5 k	N/A
Chang et al. (2011)	CC2430	1	32	0–0.4 mA	C	0.8–80	N/A
Stanslaski et al. (2012)	Activa PC + S	4	N/A	0–25.5 mA	C	2–250	N/A
Bagheri et al. (2013)	FPGA-based	256	N/A	0.02–0.25 mA	O	0.5–500	92.8%
Chen et al. (2014)	BSP	8	81.92	0–0.03 mA	C	0.8–7 k	N/A
Lin et al. (2014)	N/A	N/A	25	0–0.04 mA	O	N/A	N/A
Liu et al. (2014)	PennBMBI	4	N/A	0–1 mA	C	300–6 k	N/A
Shoaran et al. (2015)	FPGA	16	N/A	N/A	C	30–1.7 k	N/A
Lin et al. (2016)	8051	1	10	8.25–229 μA	O	N/A	N/A
Do Valle et al. (2016)	N/A	8	N/A	N/A	C	0–500	N/A
Irwin et al. (2016)	ATMega 328p	16	8	N/A	C	0.1–20 k	N/A
Kassiri et al. (2017b)	GL060V5	24	10	0.01–1 mA	C	10–5 k	N/A
Xie et al. (2017)	CC2541	32	32	N/A	C	0–7.5 k	N/A
Cheng et al. (2018)	YBSP	16	N/A	0.5–3 mA	C	0.59–117	N/A
Hügle et al. (2018)	MSP430FR5994	N/A	8	N/A	C	N/A	N/A
Li and Cook (2018)	N/A	N/A	N/A	1–10	O	0–130	ANT: 46–90%; HC: 48–95%
Kassiri et al. (2019)	AGL 060V5	16	32	0.05–10 mA	O	N/A	N/A
Pazhouhandeh et al. (2019)	N/A	64	10	0–3 mA	C	0.1–5 k	N/A
Lee et al. (2020)	ATUC3C2256C	8	N/A	0–0.51 mA	C	0–150	N/A

*ANT, anterior thalamus; C, closed-loop; HC, hippocampus; N/A, not available; O, open-loop; VNS, vagus nerve stimulation.*

On the other hand, the main motivation of closed-loop stimulation is minimization of treatment side effects by providing only the necessary stimulation required within time window, as determined from a guiding marker. The closed-loop stimulation usually uses a lower intensity and in turns limits any unwanted direct stimulation of nearby fiber tracts, such as those in the internal capsule for STN stimulation. Closed-loop stimulation could be essential not only to reverse direct side effects of stimulation, but also to minimize adverse effects due to combined pharmacological treatment as dyskinesia of dopaminergic medication in PDs (Arlotti et al., 2018). Adverse effects of DBS on sleep might decrease during a closed-loop stimulation of ANT for treatment of epilepsy (Voges et al., 2015).

Several kinds of closed-loop configurations are proposed. Firstly, closed-loop stimulation used feedback from peripheral signals, such accelerometers and/or electromyogram, are applied to automatically determine stimulation timing or intensity. For example, resting tremor is easily recorded using accelerometers providing potential source of feedback to modulate DBS (Cagnan et al., 2017). The scenario using accelerometers is also working in closed-loop seizure control (Chang et al., 2011). Secondly, closed-loop stimulation used local field potentials sensing from the same or nearby electrodes to automatically determine stimulation timing or intensity. For example, beta band (∼20 Hz) of the STN can be tracked at the site of stimulation in PDs (Little et al., 2016). Thirdly, closed-loop stimulation used ECoGs sensing from the cortex to automatically determine stimulation timing or intensity. Gamma activity and beta activity of the motor cortex in the closed-loop stimulation are used to the control of dyskinesias (Swann et al., 2018) and tremor (Herron et al., 2016), respectively. The closed-loop control used DBS of the zona incerta regarding to cortical epileptiform activity is demonstrated to stop seizures in rats (Young et al., 2011).

In patients with epilepsy or PD, many brain areas appear rhythmic activity. In addition to provide an amplitude-related feedback in the closed-loop system, fluctuations in activity timing (i.e., phase coupling) and/or strength (amplitude coupling) are crucial for network operation within different brain regions. Stimulation at a certain phase of neural activity can disrupt synchrony. This phase-specific DBS has shown to be effective in acutely suppressing tremor in a group of patients with essential tremor using ∼40% of total electrical energy associated with conventional high-frequency DBS (Cagnan et al., 2017). This stimulation approach has the potential to minimize DBS-induced side effects by reducing the amount of energy delivered into the brain.

In addition, adapting DBS is also proposed and characterized two approaches (Rosa et al., 2015). One is a binary approach with effective stimulation wither on or off. The other approach is a scalar method with stimulation voltage being varied up to therapeutic values. The stimulated voltage is not rapidly increased. For the binary on-off stimulation, it is managed by the incorporation of a ramping of stimulation onset and offset. With regard to the scalar stimulation approach, the stimulating value at sub-threshold voltages remains to be clarified. Consideration of patient behavior, such as sleep or walking, could also further aid in determining optimal stimulation patterns. For example, high-frequency stimulation at a certain period of the decision-making process impaired patient’s behavior, suggesting that adapting stimulation timing according to patient behavior could limit such adverse effects (Herz et al., 2018). In summary, these observations highlight a potential new way for stimulation control. Tailoring stimulation is not only according to pathology and its circuit manifestations, but also according to everyday actions and behaviors of patients.

The present study has collected numerous literatures for DBS technology development in the open-loop and closed-loop manners on PD (Table 3) and epilepsy (Table 4). Most of studies in the open-loop architecture have described DBS effect on symptom reduction regarding to different stimulation sites. Clinical evaluation for the open-loop DBS effect on several PD symptoms regarding different stimulation sites has been reviewed previously (Benabid et al., 2009; Wong et al., 2019). More information of neurophysiological aspects and stimulation sites for open-loop DBS on epilepsy control has been reviewed elsewhere (Yan et al., 2019; Zangiabadi et al., 2019). The closed-loop stimulation system provides relatively limited information on long-term DBS effect yet. It may raise more attention in future evaluation of the closed-loop DBS in clinic.

### Signal Processing Unit

**FIGURE 2:**
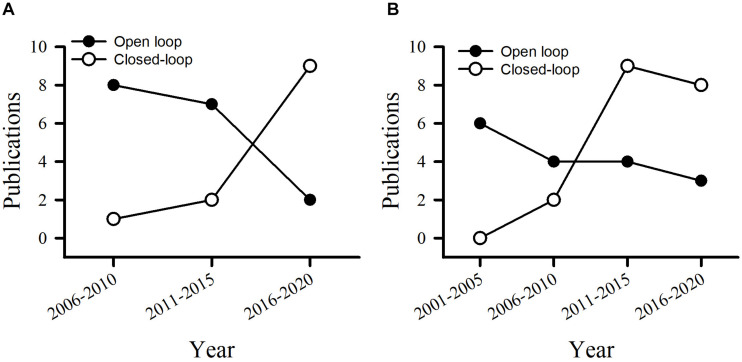
Publications utilizes the open loop and closed-loop architecture in the control of PD **(A)** and epilepsy **(B)**.

For patients with PD or epilepsy, there is two major divisions for system architectures: open loop and closed-loop. In this section, we introduce development of system in terms of platform, recording channel, stimulation intensity and architecture, and verification of animal or humans. Table 3 lists studies utilized in PDs. Table 4 summarizes studies of epilepsy. For the open-loop architecture, the signal processing unit is emphasized on signal recording and analysis which is separated from stimulation. In a closed-loop architecture, the signal processing unit and stimulation is interacted each other. As shown in the bottom panel of Figure 1, the closed-loop system exhibits a timing-schedule processing from recording, feature enable (by threshold of data length or particular waveform amplitude), feature extraction, and classification. From system development viewpoint, system development for the open loop configuration is progressively decreased (Figure 2). Instead, studies of the closed-loop architecture have increased recently in the application of PD (Figure 2A) and epilepsy (Figure 2B). The closed-loop concept for epilepsy seems to be earlier than PD studies, which reflects more available closed-loop commercial device for epilepsy compared with PD (Tables 1, 2).

There are two major streams for signal processing. One focuses on process and stimulation at the same site or neighboring area. The other is recording and processing of multiple brain regions and/or other accessories which is away from the stimulation region. For the first design, small amount of recording channels is needed and usually integration of recording and stimulation electrodes together. In this system configuration, studies often use features from a local brain region, such as STN or GPi for PD and hippocampus for temporal lobe epilepsy. It has an advantage of better understanding for characteristics of this local brain region in response to PD or epilepsy. However, neurodegeneration within this local region may result in progressive reduction of therapeutic effect. For the second system configuration, these studies have a great capacity to record and analyze signals from bulks of field potentials or neuronal activities from various brain regions. Increased channels and memory with a fast system clock is a crucial requirement. Recently, hundreds of recording electrodes coincident with efficient channel architecture for amplifier and analysis have been developed (Kassiri et al., 2017a; Zhou et al., 2019).

Two major signal processing systems [i.e., microcontroller unit (MCU) and system on a chip (SoC)] are developed. At the early stage, MCU has been developed and widely used since 1980s. The MCU system generally consists of four parts: a central processing core, program storage memory, data storage memory and one or more timers/counters with different resolutions. For example, a mobile single-channel wireless closed-loop epileptic seizure detector uses Texas Instrument’s CC2430 module as the MCU and has demonstrated its advantage on seizure control in freely moving rats (Young et al., 2011). To reduce power consumption and minimize the size of the system, configuration of system-on-chip (SoC) has been developed for years. A SoC contains a MCU, a flash memory, necessary capacitors, resistors, oscillators and other components, making it ultra-small and low power consumption. Detail SoC architecture can see in previous reviews (Wu et al., 2018; Yang and Sawan, 2020).

In a closed-loop architecture, feature extraction process with effective classification algorithm plays an important role in attaining efficient control of PD symptoms or seizures. The goal of the feature extraction process is to derive a biomarker from electrophysiological or behavioral signals that are unique during the defined state but not occurring at other states. Frequency domain features are the most commonly used features in previous studies of both PD (Table 5) and epilepsy (Table 6). Available features in the frequency domain are power or amplitude of particular bands, discrete wavelet analysis for instantaneous power of particular bands, etc. Movement event-related potential has been transferred into 17-dimensional features (Meyer wavelet scales: 9–27) to quantify 13–30 Hz in PD (Loukas and Brown, 2012). Beta frequency power is mostly used as biomarker for the control of PD symptom (Table 5). In contrast, frequency powers of various bandwidths are used for seizure detection or prediction in a closed-loop architecture (Table 6). Multiple features, such as approximated entropy and coastline, are used in the control of epilepsy.

**TABLE 5:** The closed-loop stimulation system for PD.

Source	Platform	Clock (MHz)	Filter (Hz)	Feature
Lee et al. (2008)	N/A	0.2	17–5.3 k	Amplitude of 10–50 Hz
Azin et al. (2011)	N/A	1	0–10 k	Amplitude of 20–40 Hz
Loukas and Brown (2012)	NI DAQ	N/A	N/A	Meyer’wavelet for instantaneous bandpower (13–30 Hz)
Parastarfeizabadi et al. (2016)	MCU	N/A	0–130	Amplitude of 13–35 Hz
Arlotti et al. (2016)	MSP 430	N/A	N/A	Bandpower (10–16 Hz)
Herron et al. (2016)	Activa PC + S	N/A	N/A	Bandpower (20–32 Hz)
Little et al. (2016)	N/A	N/A	N/A	Bandpower (12–30 Hz)
Wu et al. (2017)	IEC 60601-1	13.56	12–30	Bandpower (4–64 Hz) and entropy
Swann et al. (2018)	Activa PC + S	N/A	60–90	Bandpower (80 ± 2.5 Hz)
Chen et al. (2018)	FPGA	N/A	30–10 k	Amplitude of 20–40 Hz
Zhou et al. (2019)	WAND	166	1–200	Bandpower (0–4 and 4–7 Hz)
Xu et al. (2020)	NI DAQ	N/A	1–8 k	AP probability mapping

*AP, action potential; N/A, not applicable.*

**TABLE 6:** The closed-loop stimulation system for epilepsy.

Source	Platform	Clock (MHz)	Filter (Hz)	Feature
Avestruz et al. (2008)	N/A	N/A	0–500	Bandpower (15–40 Hz)
Halpern et al. (2009)	RNS	N/A	1–333	Bandpower (1–4, 4–8, 8–13, 13–25, and 20–50, > 50 Hz), total power, spike
Chen et al. (2011a)	FPGA	402	N/A	Bandpower (7–9 and 14–18 Hz) and ApEn
Chen et al. (2011b)	FPGA	402	0–3.2 k	Bandpower (7–9 Hz and harmonics) and ApEn
Young et al. (2011)	CC2430	32	0.8–72	Bandpower (7–9 and 14–18 Hz) and ApEn
Zanos et al. (2011)	Neurochip-2	N/A	0.5–5 k	Bandpower (10–20 Hz)
Chang et al. (2011)	CC2430	32	0.8–72	Bandpower (10–20 Hz)
Stanslaski et al. (2012)	Activa PC + S	N/A	0–250	Bandpower (beta band, ∼80 Hz)
Chen et al. (2014)	BSP	3.125	0.8–7 k	entropy and Bandpower (0.8–10 Hz)
Liu et al. (2014)	PennBMBI	N/A	300–6 k	Spike detection
Shoaran et al. (2015)	FPGA	N/A	30–1.7 k	Channel-based coastline features (linelength)
Do Valle et al. (2016)	N/A	8	0–500	Bandpower (0.5–4,:4–8,:8–13, and 13–30 Hz)
Irwin et al. (2016)	ATMega 328p	8	0.1–20 k	Bandpower (75–150 Hz)
Kassiri et al. (2017b)	GL060V5	10	10–5 k	Magnitude and Phase of FIR and Hilbert filter
Xie et al. (2017)	CC2541	32	0–7.5 k	Spike amplitude > 150 μV
Cheng et al. (2018)	BSP	N/A	0.59–117	Bandpower (7–9 and 14–18 Hz)
Hügle et al. (2018)	MSP430FR5994	8	N/A	Intrinsic mode functions
Pazhouhandeh et al. (2019)	N/A	10	0.5–10 k	Bandpower and heart rate
Lee et al. (2020)	MCU	2	0–150	ApEn and power (5, 10, and 15 Hz)

*ApEn, approximate entropy; FIR, finite impulse response.*

In addition to feature extraction, various classification methods, such as support vector machine, regression classifiers, linear square classifier, etc., have been used in previous studies (Zhou et al., 2019; Yang and Sawan, 2020). Recently, convolutional neural network (CNN) is composed of convolution, pooling and fully connected layers. Currently, most CNN algorithms are higher complexity and executed using CPU or GPUs. A network architecture called SeizureNet on a low-power processing microcontroller unit to predict seizure (Hügle et al., 2018). There is another low-power CNN processor (TrueNorth developed by IBM) has been used for seizure detection (Merolla et al., 2014). Other detail classifiers in a closed-loop architecture has been reviewed elsewhere (Yang and Sawan, 2020).

## Discussion and Future Prospects

We are witnessing a rapid expansion in the development of implantable DBS devices for clinical uses. Because of their complexity they are classified by regulatory bodies into the highest risk category for implantable device (Class III) and are required to complete a very rigorous regulatory approval process before clinical use. Preclinical studies (including animal verification) form an important component of this approval process. The present review describes various components in the open loop and closed-loop configuration that are available to researchers when considering to demonstrate device safety and effectiveness.

In the near future, trends of automation and effective information processing, as well as device miniaturization are anticipated. In addition to advanced development of circuit and IC production, low-noise and low-power chopping amplifier and SoC is existing. Numerous systems that can record EEG, ECoG or neuronal activities have increasingly reliable and accurate detection or prediction algorithms. These systems exhibit a great increased capacity for channels and memory size. A closed-loop configuration for DBS or stimulation of the cortex or peripheral nervous system expresses numerous advantages, including minimal adverse effect, reduced potential damage, increased battery life, and preserved daily regular activity. Rechargeable neurotherapy systems are more economical and lower complication than non-rechargeable devices (Chwalek et al., 2015). The main challenge in the design of rechargeable implantable devices is how to efficiently recharge the implantable battery and avoid highly increased temperatures during the charging process, which may cause skin burns.

The present study has shown an important trend for the closed-loop DBS in PD and epilepsy. Spatial selectivity is enhanced through higher resolution electrodes to increase accuracy. The closed-loop DBS is away from monotonic high-frequency stimulation and advocates dynamic stimulation in response to valuable features. These development directs us closer to the individual therapy that tracks clinical state. However, more sophisticated control requires a greater understanding of pathophysiology to allow the development of useful biomarkers and dictate stimulation. Numerous features are considered as biomarkers compared with a healthy control. It raises a problem about feature reliability throughout the entire DBS progression. Effective DBS alters brain activity progressively then leads to reduction of sensitivity and specificity in electrophysiological feature characteristics and anatomical alteration (Little and Brown, 2012). Meanwhile, intra-subject and inter-subject variability occurs in our day-by-day conditions (Saha and Baumert, 2020). Secure telemetry allows patients continuous wireless upload of data, which would allow more continuous patient assessments and more intricate control using distributed cloud computing system. At the same time, such a system integrates data from other sensors to provide summaries that aid decision-making and prevent clinicians from being overloaded from intensive information. Thus, adapting control algorithms should mature while maintaining tractability.

In the long-term, it is likely that brain stimulation therapies will be disrupted by advancing technology. For example, minimally invasive methods such as transcranial ultrasound are enabling non-invasive ablation of neural circuits for tremor (Lipsman et al., 2013). It may provide many DBS benefits without requirement of cranial surgery 1 day. A hybrid method combined distributed ultrasound systems replacing physically tethered leads may enable a considerable neural interfere to create similar DBS advantages.

## Author Contributions

Y-CW, S-FL, and F-ZS involved in project administration, study conceptualization, designed the manuscript, and assessed the literature. Y-CW and F-ZS wrote the first draft of the manuscript. Y-CW, W-HY, and F-ZS edited the subsequent drafts and revisions. All authors contributed to the article and approved the submitted version and involved in the supply of the materials.

## Conflict of Interest

The authors declare that the research was conducted in the absence of any commercial or financial relationships that could be construed as a potential conflict of interest.
